# Supplementary material for: EnzML: multi-label prediction of enzyme classes using InterPro signatures
Source: BMC Bioinformatics. 2012 Apr 25;13:61. doi: 10.1186/1471-2105-13-61 (PMC3483700; doi:10.1186/1471-2105-13-61)
Supplement: Addtional file 5 — The Java code to format the data files, evaluate and predict. The file enzml_java_code.tar.gz contains the Java code used to format database data to ARFF and XML formats, to execute cross and train-test (jackknife) evaluations and to record evaluation results to database. More information is included in the readme.txt file and the Javadoc files. The code can be used with a MySQL database. To use a different database software, other JDBC drivers might be required. [file 1471-2105-13-61-S5.gz › java_code/utils/doc/uk/ac/ed/inf/utils/database/class-use/DbReader.html]

Uses of Class uk.ac.ed.inf.utils.database.DbReader


---


|  |  |  |
| --- | --- | --- |
| **Overview** \| **Package** \| **Class** \| **Use** \| **Tree** \| **Deprecated** \| **Index** \| **Help** | |  |
| PREV   NEXT | **FRAMES**    **NO FRAMES**     **All Classes** |  |


---


## **Uses of Class uk.ac.ed.inf.utils.database.DbReader**

| Packages that use DbReader | |
| --- | --- |
| **test.database** |  |
| **uk.ac.ed.inf.utils.database** |  |

Uses of DbReader in test.database

| Methods in test.database that return DbReader | |
| --- | --- |
| `static DbReader` | `DbReaderTest.getDbReader()` |

Uses of DbReader in uk.ac.ed.inf.utils.database

| Methods in uk.ac.ed.inf.utils.database that return DbReader | |
| --- | --- |
| `DbReader<java.lang.String,java.lang.String>` | `DbManager.getDbReader()` |

---


|  |  |  |
| --- | --- | --- |
| **Overview** \| **Package** \| **Class** \| **Use** \| **Tree** \| **Deprecated** \| **Index** \| **Help** | |  |
| PREV   NEXT | **FRAMES**    **NO FRAMES**     **All Classes** |  |


---
